# Biological Synthesis of Genistein in *Escherichia coli*

**DOI:** 10.4014/jmb.1911.11009

**Published:** 2019-12-23

**Authors:** Bong-Gyu Kim

**Affiliations:** Department of Forest Resources, Gyeongnam National University of Science and Technology, Jinju 52725, Republic of Korea

**Keywords:** Isoflavone, co-culture, biotransformation, genistein

## Abstract

Genistein is a type of isoflavonoid found predominantly in leguminous plants. Genistein has diverse biological activities, such as anthelmintic and antioxidant effects, as well as inhibitory effects on the growth of several cancers. In addition, genistein is well known as a phytoestrogen. In this study, we attempted to biologically synthesize genistein from either *p*-coumaric acid or naringenin using *Escherichia coli* as a biotransformation host. Four genes, *Os4CL, PeCHS, RcIFS*, and *OsCPR*, were used for genistein production. To functionally express RcIFS and OsCPR, two members of the cytochrome P450 family, in *E. coli*, the membrane-binding anchor domain of each gene was removed, and *RcIFS* and *OsCPR* were translationally fused to generate an *RcIFS-OsCPR* hybrid. Os4CL and PeCHS, or the RcIFS-OsCPR hybrid, were then transformed into *E. coli* BL21(DE3). Using these strains, we optimized our culture system at a laboratory scale in terms of the cell density, concentrations of substrate and isopropyl-*β*-D-thiogalactoside, temperature, and culture medium. Under the optimized culture conditions, genistein was produced at up to 35 mg/l and 18.6 mg/l using naringenin and *p*-coumaric acid, respectively.

## Introduction

Isoflavones are a subclass of flavonoids frequently found in the plant kingdom. They are found predominantly in legumes and occasionally in other plants [1]. In leguminous plants, isoflavones not only act as signaling molecules mediating plant-microbial interactions with microbes such as nitrogen-fixing bacteria and rhizobia, but are also involved in defense mechanisms against pathogens and UV damage [2-4]. Isoflavones have received much attention due to their health-promoting effects, such as the prevention of cardiovascular disease and hormone-related cancers, as well as reducing the risk of type 2-diabetes, Alzheimer’s disease, and osteoporosis [5-7]. The major source of isoflavones with these diverse biological activities is soybeans. The isoflavone content of soybeans was reported to be 1.26 mg/g, including 0.56 mg/g daidzein and 0.25 mg genistein [1]. Generally, soybean is consumed in processed food products such as tofu, soymilk, soybean paste, and cheonggukjang. The isoflavone content in these foods was reported to be 0.01–0.12 mg/g for soymilk, 0.82 mg/g for soybean paste, and 0.40 mg/g for cheonggukjang [8]. Unfortunately, soybeans not only produce isoflavones at very low levels, but also display seasonal and geographic variations in their isoflavone contents. In addition, because soybeans produce many phytochemicals in addition to isoflavones, the use of soybean-derived isoflavones for industrial purposes requires complex separation and purification processes. For these reasons, there are many barriers to exploiting isoflavones for their biological functions and to applying them in industrial processes.

Microorganisms can be used as a versatile alternative tool for the production of valuable natural compounds [9-12]. Early studies used microorganisms to produce valuable compounds from simple substrates by introducing one or two plant biosynthetic genes [13-15]. This method has been used for the biosynthesis of many important factors. Recently, there have been major efforts to biosynthesize plant-derived compounds from simple carbon sources such as glucose using single microorganism strains [16-18]. However, the titers of natural products biosynthesized from these simple carbon sources were very low. To overcome this obstacle, metabolically engineered microorganisms have been developed to increase the synthesis of these beneficial compounds through the stable supply of co-factors or co-substrates [19]. Using these approaches, many important compounds, such as flavonoids, terpenoids, and alkaloids, have been successfully biosynthesized using microorganisms, most commonly *Escherichia coli*. However, the use of a single strain generated by metabolic engineering can lead to severe metabolic imbalances during the biosynthesis of valuable compounds, making it difficult to increase the yields of the final biosynthesis product. Recently, co-culture systems using two or more strains have been developed to overcome these problems and increase the final titers [20, 21].

Genistein is a well-known isoflavone compound with diverse medical benefits such as anthelmintic and antioxidant effects, as well as inhibitory effects on prostate, brain, cervix, breast, and colon cancers [1, 7]. In particular, genistein is commonly used as a phytoestrogen that exerts biological effects via the estrogen receptor [7]. It is biosynthesized through the branch pathway of the phenylpropanoids pathway, and is found primarily in legumes. The biosynthesis of genistein in legumes begins with the production of cinnamic acid through the deamination of phenylalanine by phenylalanine ammonia lyase (PAL). Cinnamic acid is converted to naringenin through the sequential activity of four proteins: cinnamate-4-hydroxylase (C4H), 4-coumarate-CoA ligase (4CL), chitin synthase (CHS), and endochitinase (CHI). Naringenin is used for the biosynthesis of diverse flavonoids, including isoflavones [22]. The key protein for genistein biosynthesis is isoflavone synthase (IFS), which catalyzes the production of 2,5,7,4’-tetrahydroxy isoflavone by migrating the B ring of naringenin from C2 to C3. Then, 2,5,7,4’-tetrahydroxy isoflavone is converted to genistein spontaneously or by the activity of 2-hydroxyisoflavanone dehydratase [22, 23]. IFS is a member of the cytochrome P450 protein family, and is difficult to functionally express in *E. coli* due to its hydrophobic membrane-bound anchor domain, which gives it low solubility. Furthermore, for IFS to function, an electron from NADPH must be provided by cytochrome P450 reductase (CPR). CPR also has a hydrophobic membrane-bound anchor domain. Previously, the hydrophobic domains of both IFS and CPR were removed to functionally express the proteins in *E. coli*; this led to the successful production of genistein from naringenin. However, the final yield was very low [24].

In this study, we used four genes, *4CL, CHS, IFS*, and *CPR*, to synthesize genistein from *p*-coumaric acid in *E. coli*. To develop a simplified fermentation protocol, we optimized the culture conditions in terms of media, temperature, substrate feed concentration, isopropyl-*β*-D-thiogalactoside (IPTG) concentration, and cell density.

## Materials and Methods

### Bacterial Strains, Vectors, Reagents, and Media

*E. coli* DH5α was used for gene cloning and plasmid amplification, and *E. coli* BL21 (DE3) was used as a biotransformation host. Reagents including *p*-coumaric acid, naringenin, and genistein were purchased from Sigma Aldrich (USA). The pGEX 5X-3 and pETDuet vectors were used to construct the genistein biosynthetic pathway from *p*-coumaric acid. IPTG was obtained from Bioneer Corporation (Korea). Terrific broth (TB), Luria broth (LB), and yeast extract peptone dextrose broth (YPD) were purchased from Difco (USA). Unless otherwise stated, glucose and glycerol were supplied in the culture medium at concentration of 20 g/l.

### Constructs

*Os4CL* from *Oryza sativa* was amplified using HotStart Taq DNA polymerase (Qiagen, Germany) using a previously constructed plasmid [25] as a template with 5’-ATGGATCCGATGGGGTCGGTGGCGGCGG-3’ as a forward primer (BamHI site is underlined) and 5’-ATGCGGCCGCTTAGCTGCTTTTGGGCGC-3’ as a reverse primer (NotI site is underlined). The PCR product was gel-purified, digested with BamHI and NotI, and cloned into the corresponding pETDuet sites. To clone *PeCHS* from *Populus euramericana*, PCR was performed using a previously constructed plasmid [26] as template with HotStart Taq DNA polymerase and 5’-ATCATATGGCAC CGTCGATTGAGGA-3’ as a forward primer (NdeI site is underlined) and 5’-ATGGTACCTCATGAGTAA ATTGTTTGTTCT-3’ as a reverse primer (KpnI site is underlined). The PCR product was digested with NdeI and KpnI and subcloned into the second multiple cloning site of pETDuet containing the *Os4CL* gene at the first multiple cloning site and named pE-C4. Finally, the *RcIFS-OsCPR* fusion gene construct using RcIFS from *Trifolium pretense* and OsCPR form *O. sativa* was cloned as previously described to construct the E-IFS strain [24].

### Biotransformation and Analysis of the Reaction Product

A single colony of transformants was inoculated in LB medium supplemented with 100 μg/ml ampicillin and grown at 37°C overnight with shaking at 200 rpm. The next day, 250 μl seed culture was transferred to a 250-ml flask containing 25 ml fresh LB medium supplemented with 100 μg/ml ampicillin. The flask was cultured at 37°C and 200 rpm until it reached an optical density at 600 nm (OD_600_) of 0.6. Next, IPTG was added at a final concentration of 0.1 mM. The flask was then incubated at 25°C for 20 h at 200 rpm to induce the expression of the recombinant proteins. *E. coli* cells were collected by centrifugation at 4,000 rpm and 4°C, and then washed twice in Andrew’s Magic Media (AMM) [27]. The cells were resuspended at OD_600_ = 3.0 in 25 ml AMM containing 100 μg/ml ampicillin, 2% glycerol, and 0.1 mM IPTG. The flask was then incubated at 25°C for 12 h for biotransformation. The reaction products were extracted from the culture medium with the same volume of ethyl acetate. The upper layer was dried with a speed vacuum drier, dissolved in 60 μl dimethyl sulfoxide (DMSO), and used for high-performance liquid chromatography (HPLC) (Varian Medical Systems, USA) equipped with a photodiode array detector and polar C18 reversed-phase column (Agilent Technologies, Santa Clara, CA, USA; 3.5 μm, 4.6 × 250 mm). The mobile phase contained water (solvent A) and acetonitrile (solvent B) with 0.1% formic acid at a flow rate of 1 ml/min. UV detection was dually monitored at 270 and 290 nm. HPLC analysis was performed according to the following program: 20% solvent B at 0 min, 45% solvent B at 10 min, 60% solvent B at 40 min.

## Results and Discussion

### Genistein Production from Naringenin

**Fig. 1:**
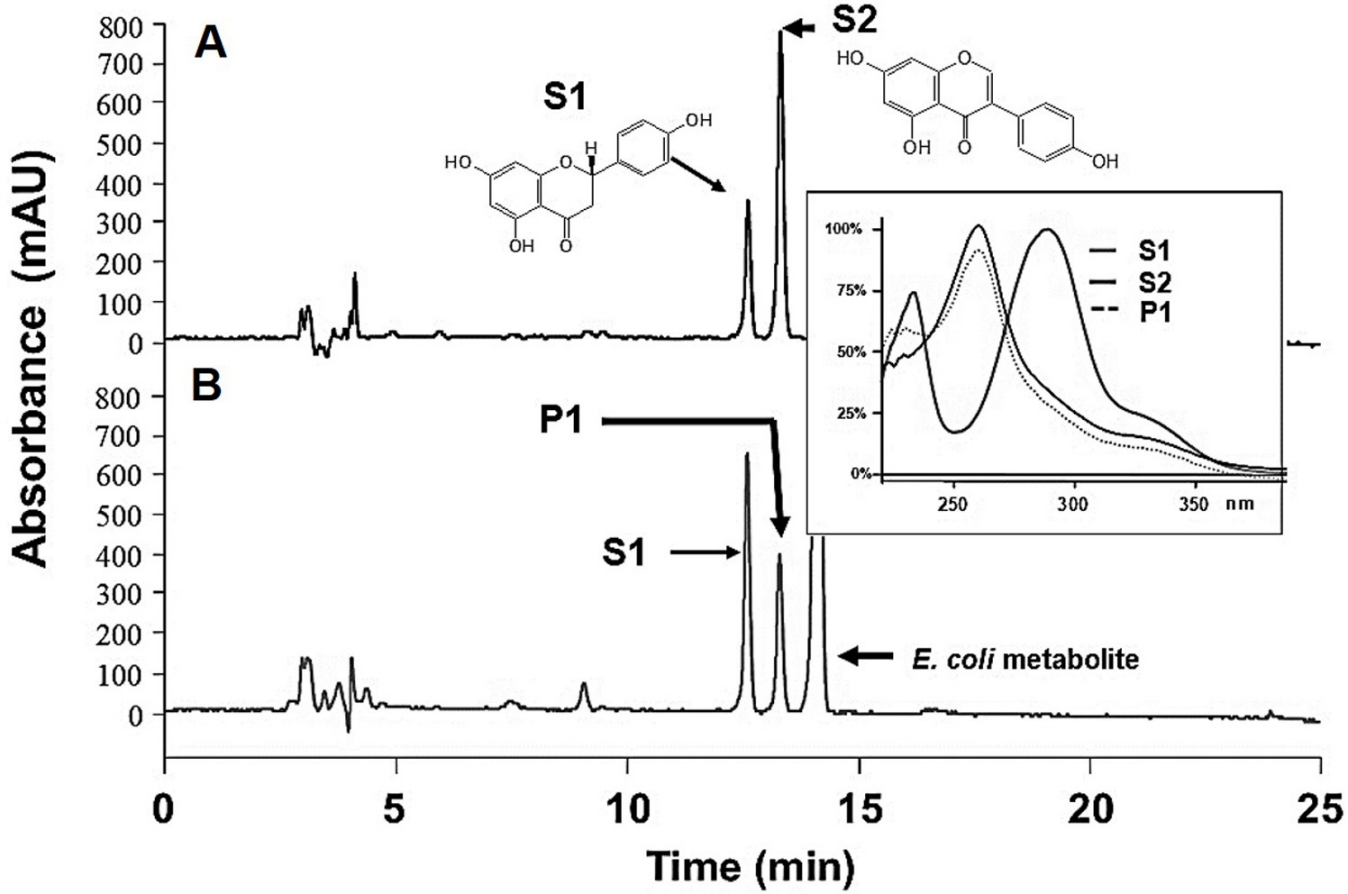
High-performance liquid chromatography analysis of genistein biosynthesized from naringenin using *E. coli* BL21(DE3) transfected with *RcIFS-OsCPR*. (**A**) Standard naringenin (S1) and genistein (S2); (**B**) reaction product (P1).

When IFS and CPR are introduced into *E. coli*, it is possible to biosynthesize genistein from naringenin. However, the functional expression of IFS and CPR is difficult in *E. coli* because both proteins contain membrane anchorage domains that produce insoluble proteins in *E. coli* due to the absence of an endoplasmic reticulum. Despite the difficulty in functionally expressing these genes in *E. coli*, it can be done successfully if the membrane anchorage domains are removed from both genes and the genes are transcriptionally fused. In this study, a previously developed *RcIFS-OsCPR* fusion gene construct [24] was used for genistein production from naringenin. To confirm the production of genistein from naringenin in *E. coli*, the *RcIFS-OsCPR* fusion gene was introduced into *E. coli* BL21(DE3) and designated as strain E-IFS. E-IFS was incubated at 25°C for 12 h to induce recombinant protein expression; the cultured E-IFS cells were then washed twice with AMM-glycerol. The production of genistein from naringenin in E-IFS was conducted at 30°C, and *E. coli* BL21(DE3) carrying the pGEX 5X-3 vector as a control was incubated under the same conditions. After 24 h of biotransformation, the reaction products of the E-IFS strain were analyzed by HPLC. A new peak was shown at 14.7 min (P1) that was not observed in the control strain (Fig. 1). The retention time of P1 was indistinguishable from that of authentic genistein. In addition, the UV spectrum of P1 was identical to that of standard genistein, and its molecular weight was 270 Da according to mass spectrometry. These results indicate that E-IFS successfully converted naringenin to genistein.

**Fig. 2:**
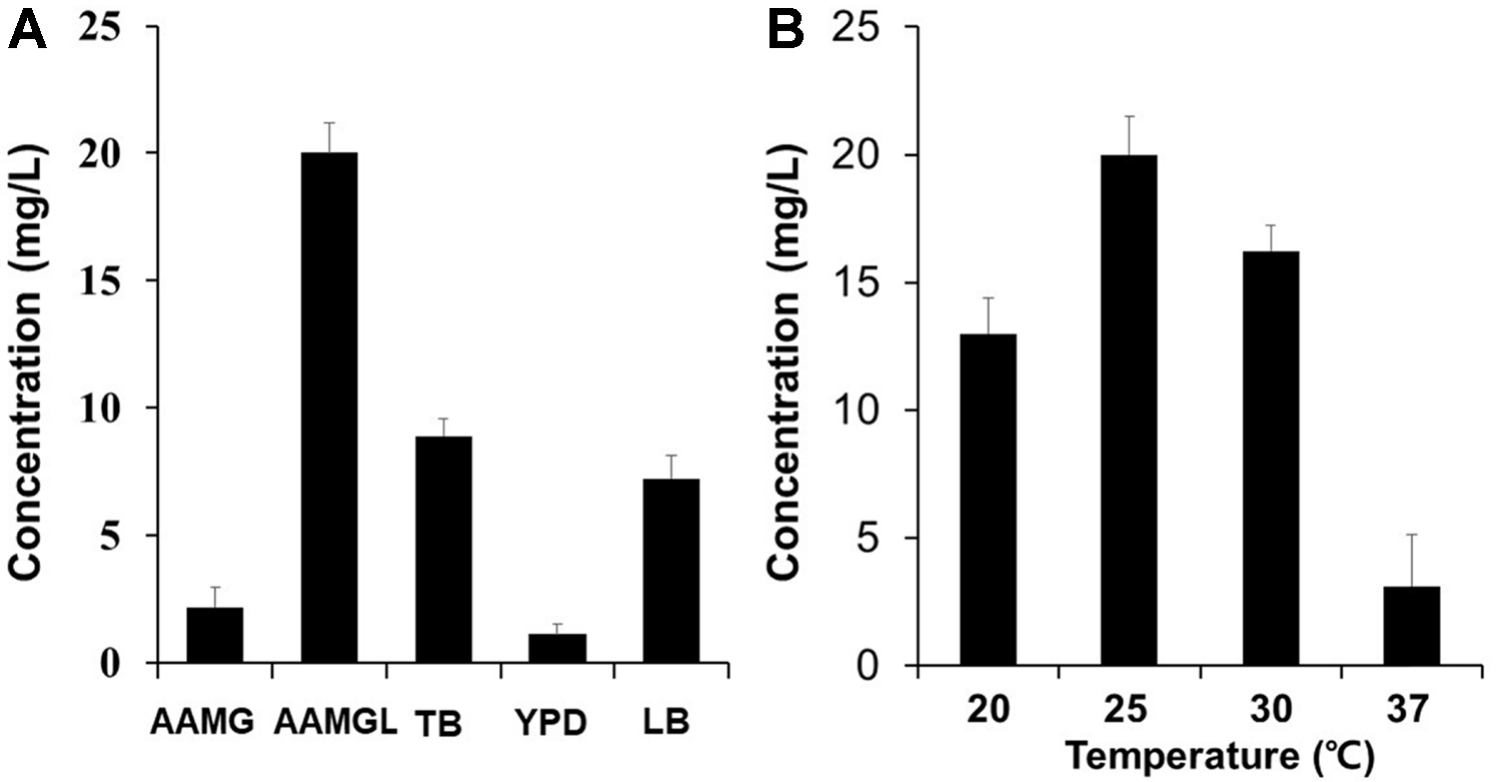
Determination of optimum culture medium (A) and temperature (B) for genistein production. Biotransformation was performed at 25°C after the induction of recombinant proteins at 25°C for 12 h using the E-IFS strain expressing RcIFS-OsCPR. AMM: Andrew’s Magic Media, AMMG: AMM-glucose, AMMGL: AMM-glycerol. Data represent the mean ± SD of three biological replicates (*n* = 3).

To begin the optimization of genistein production from naringenin, we first tested two parameters: biotransformation temperature (20, 25, 30, and 37°C) and growth medium (AMM-glucose, AMM-glycerol, TB, YPD, and LB). After inducing recombinant protein expression, cells were collected, briefly washed twice in AMM, resuspended in AMM-glycerol, and cultured at the above-mentioned temperatures and media. Among the tested media, AMM-glycerol showed the highest yield (20.5 mg/l), followed by TB (8.9 mg/l), LB (7.2 mg/l), AMMglucose (2.2 mg/l), and YPD (1.5 mg/l) (Fig. 2A). Among the tested temperatures, the highest yield was obtained at 25°C (20.5 mg/l), followed by 30°C (16.2 mg/l), 20°C (13.2 mg/l), and 37°C (3.5 mg/l) (Fig. 2B). Based on the above results, subsequent experiments were performed using AMM-glycerol and 25°C for biotransformation.

**Fig. 3:**
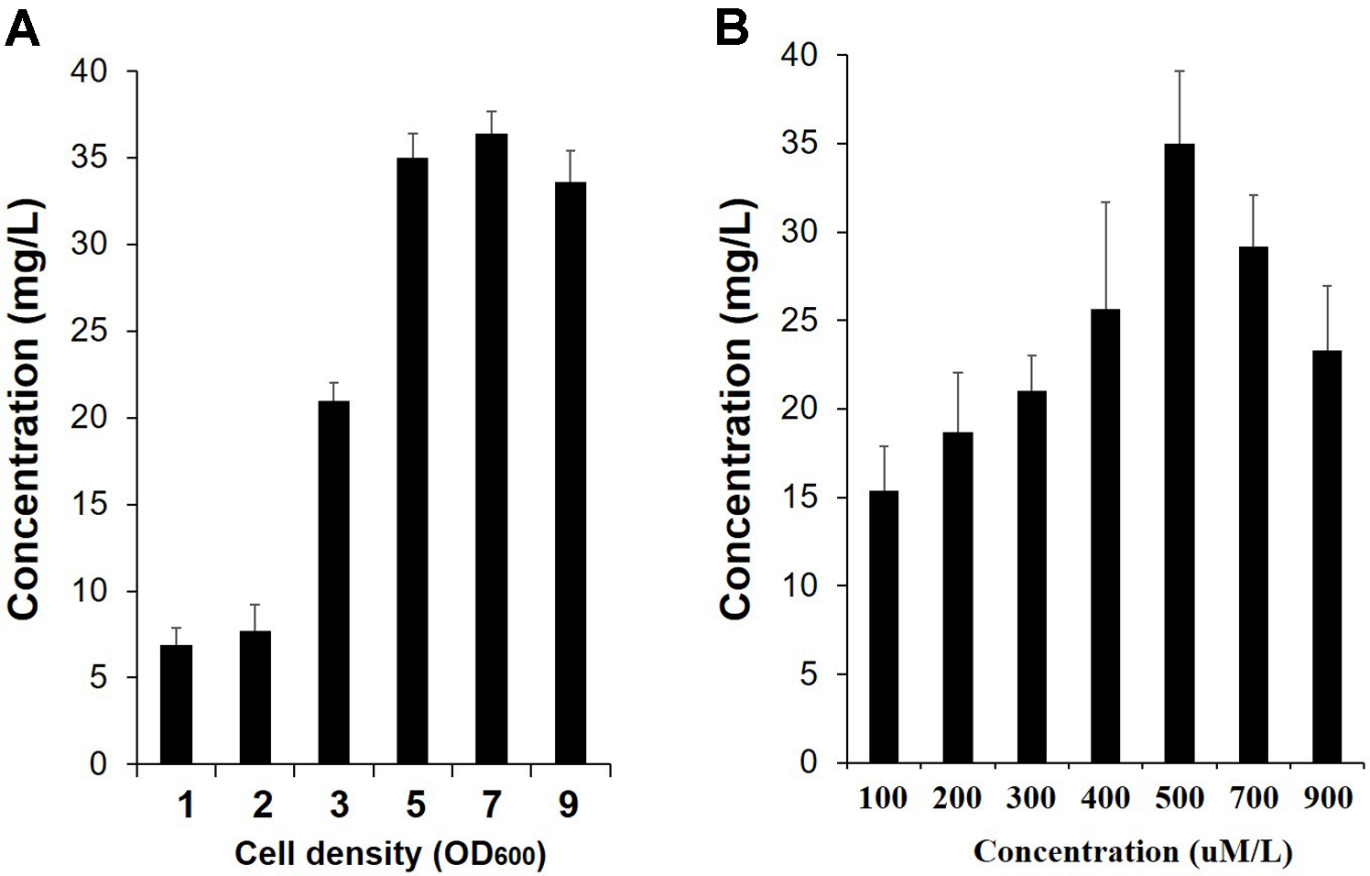
Determination of optimum cell density (A) and naringenin feed concentration (B) for genistein production. Biotransformation was performed at 25°C after the induction of recombinant proteins at 25°C for 12 h using the E-IFS strain expressing RcIFS-OsCPR. All data were obtained after incubation for 48 h at 25°C with shaking at 200 rpm. Data represent the mean ± SD of three biological replicates (*n* = 3).

Next, we determined the optimal cell concentration. Cell concentrations were adjusted to OD_600_ values of 1.0, 2.0, 3.0, 4.0, 7.0, and 9.0; the cells were then incubated with 200 μM naringenin. As the cell concentration increased, genistein production continued to increase, but the highest yield was observed at OD_600_ = 7.0, producing a yield of approximately 35 mg/l genistein (Fig. 3A). However, at low cell concentrations of OD_600_ = 1.0 and 2.0, genistein production was low.

Because high concentrations of naringenin can lead to cell growth inhibition or cause an increased metabolic burden resulting in low genistein production, we next determined the optimal substrate feed concentration (100, 200, 300, 400, 500, 700, and 900 μM/l). Among the tested naringenin concentrations, the highest yield was obtained at 500 μM naringenin.

Using these optimized biotransformation conditions, the production of genistein from naringenin was monitored for 48 h. The production of genistein began to increase after 6 h incubation, and the highest production was observed after 24 h. At this time, the yield of genistein was approximately 35 mg/l, 2.3-fold more than that previously reported (Fig. 3B) [24]. However, no increase in product was observed despite the increase in incubation time. It is likely that no increase in genistein production occurred because IFS used only the S form of naringin, while naringenin contains a mixture of both the R and S enantiomorphs [23, 24].

### Genistein Production from *p*-Coumaric Acid

**Fig. 4:**
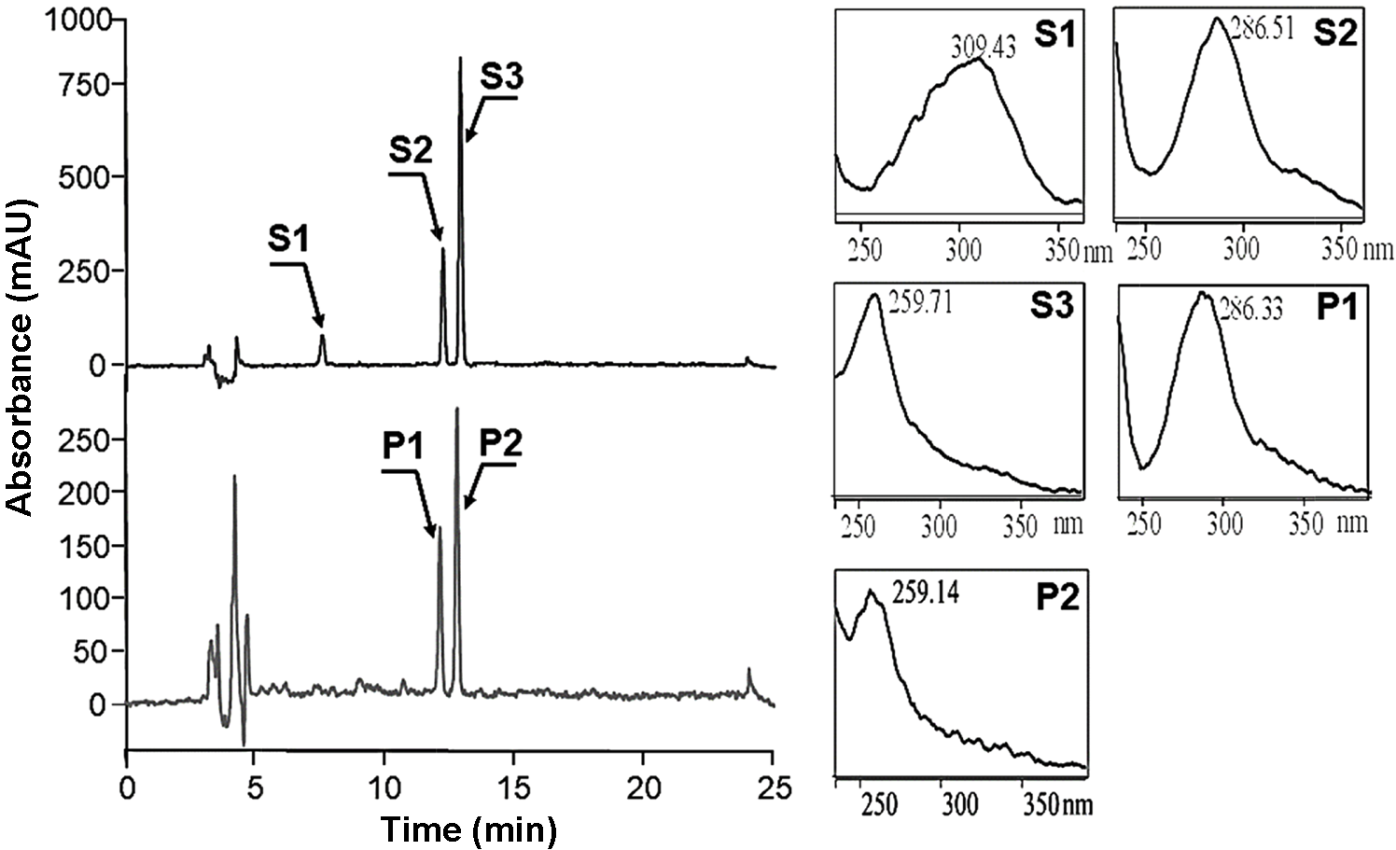
HPLC analysis of genistein biosynthesized from *p*-coumaric acid using co-culture of E-C4 and E-IFS. Biotransformation was performed at 25°C using E-C4 and E-IFS at a ratio of 1:1. S1: standard *p*-coumaric acid, S2: standard naringenin, S3: standard genistein. P1: reaction product 1, P2: reaction product 2.

Next, we attempted to produce genistein from *p*-coumaric acid, as it is less expensive than naringenin. To synthesize genistein from *p*-coumaric acid in *E. coli*, four genes, *4CL, CHS, IFS*, and *CPR*, were introduced into *E. coli* BL21(DE3) using the pE-C4 plasmid and designated as strain E-C4. E-C4 and E-IFS were grown independently overnight, collected by centrifugation, and washed twice with YM9 medium. Cells from both strains were added to the same culture flask in YM9 medium supplemented with 2% glycerol at a ratio of 1:1 (both at OD_600_ = 1.0), and then IPTG and *p*-coumaric acid were added at final concentration of 0.1 mM and 200 μM, respectively. After incubation for 48 h, the reaction products were extracted twice using the same volume of ethyl acetate and dried under a vacuum drier. The dried reaction product was then dissolved in DMSO and analyzed by HPLC. Two new peaks were observed at 13.8 min (P1) and 14.7 min (P2); these peaks were not observed in the control, which contained only the pETDuet vector (Fig. 4). P1 and P2 had same retention time and UV spectra as standard naringenin and genistein, respectively. These results indicate that this system successfully biosynthesized genistein from *p*-coumaric acid.

**Fig. 5:**
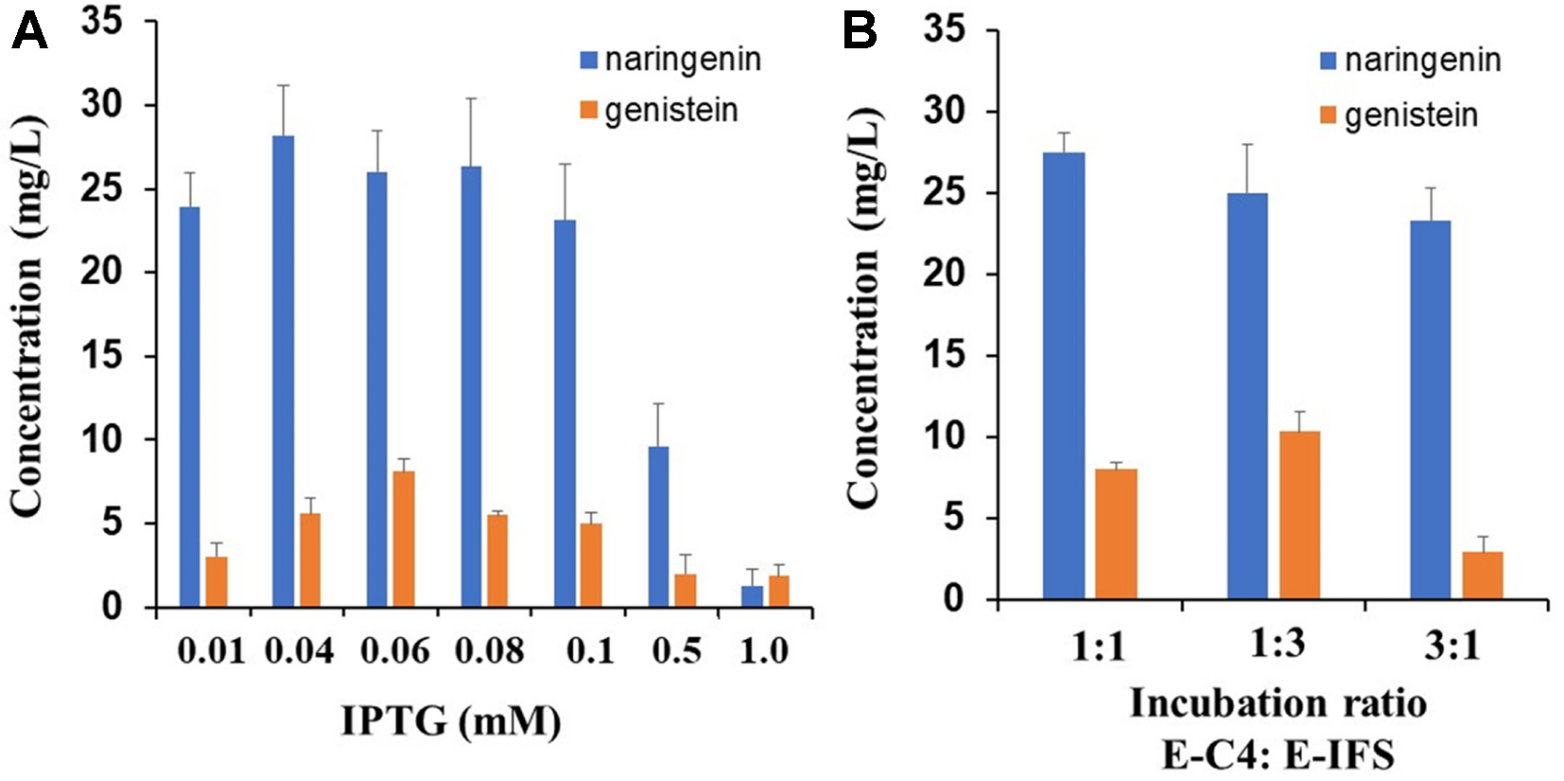
Determination of optimum IPTG concentration (A) and co-culture ratio of E-C4 and E-IFS (B) for genistein production from *p*-coumaric acid. All data were obtained after 48 h of incubation at 25°C with shaking at 220 rpm. Data represent the mean ± SD of three biological replicates (*n* = 3).

To optimize genistein production from *p*-coumaric acid, we studied the effect of different IPTG concentrations, as it is an inducer of recombinant protein expression. The results showed that protein production was strongly affected by the IPTG concentration. The highest yield (8.1 mg/l) was obtained at a concentration of 0.06 mM IPTG (Fig. 5A). However, at higher concentrations of IPTG, the biosynthesis of genistein was decreased. These results indicate that lower IPTG concentrations could reduce the protein expression burden, resulting in the increased stability of recombinant proteins and an increased conversion rate [28]. Next, the *E. coli* co-culture system was tested to determine whether different inoculation ratios of E-C4 and E-IFS would affect the production of genistein. The highest genistein production rate (10.3 mg/l) was observed in co-culture of E-C4 and E-IFS at a ratio of 1:3 after 48 h of biotransformation with *p*-coumaric acid as a substrate; however, an E-C4:E-IFS ratio of 3:1 significantly decreased both the naringenin yield and genistein production (Fig. 5B). This result suggested that a higher ratio of E-IFS is beneficial for genistein production.

**Fig. 6:**
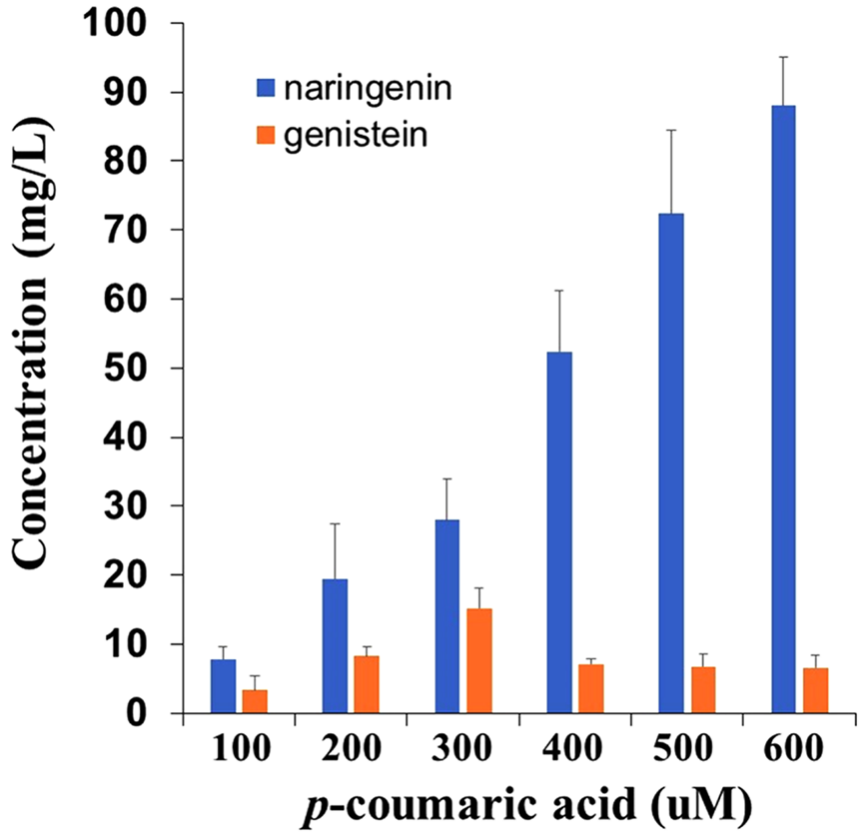
Effect of different concentrations of *p*-coumaric acid on genistein production. Cells were cultured in YM9 medium supplemented with 2% glycerol. Biotransformation was performed using E-C4 and E-IFS cells at a ratio of 1:3. All data were obtained after 48 h of incubation at 25°C with shaking at 220 rpm. Data represent the mean ± SD of three biological replicates (*n* = 3).

To determine the optimal supply concentration of *p*-coumaric acid on genistein production, cells were cultured with different concentrations of *p*-coumaric acid. After culturing for 48 h at 25°C, the reaction products were analyzed by HPLC. Among the tested concentrations, 300 μM *p*-coumaric acid showed the highest genistein production rate (15.2 mg/l), while the production of naringenin continued to increase with increasing *p*-coumaric acid concentrations (Fig. 6). These results indicate that an increase in the supply of naringenin can inhibit the biosynthesis of genistein.

**Fig. 7:**
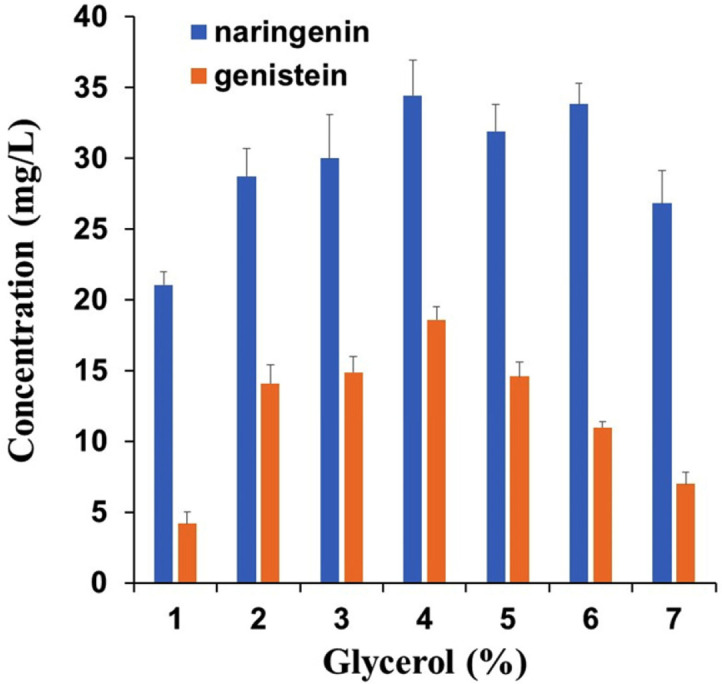
Effect of different concentration of glycerol on genistein production. Andrew’s Magic Media was used for genistein production. Biotransformation was carried out using E-C4 and E-IFS at a ratio of 1:3. The medium was supplied with 300 μM *p*-coumaric acid. All data were obtained after incubation for 48 h 25°C with shaking at 220 rpm. Data represent the mean ± SD of three biological replicates (*n* = 3).

**Fig. 8:**
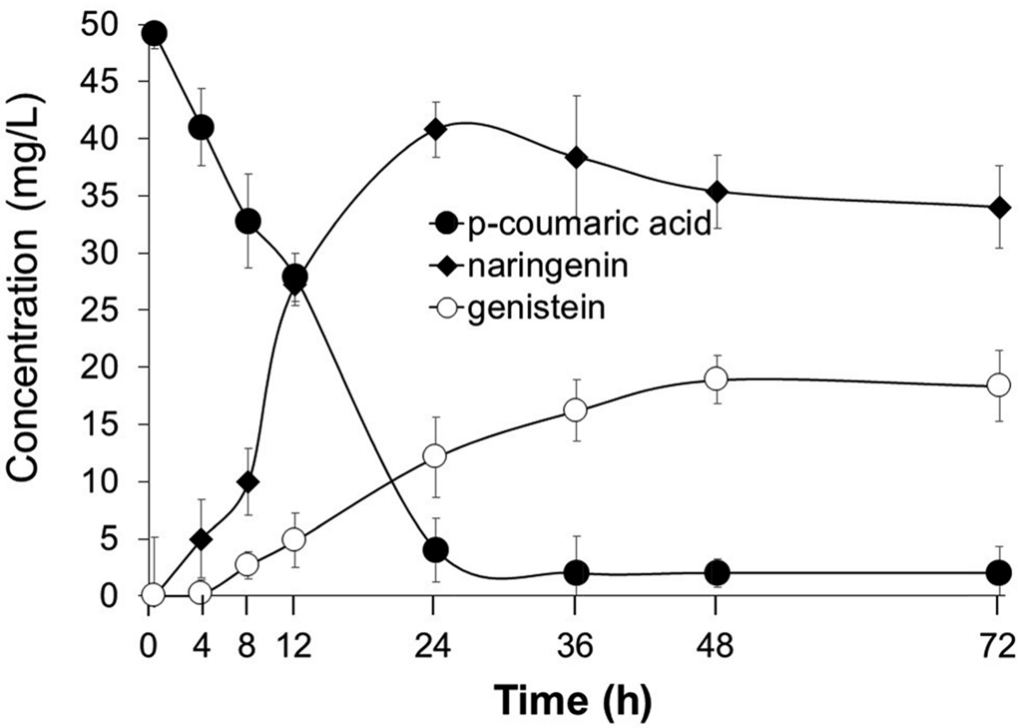
Production of genistein using E-C4 and E-IFS cells at a ratio of 1:3 and 300 μM *p*-coumaric acid. The reaction products were collected periodically and the production of genistein was monitored over 72 h. Data represent the mean ± SDof three biological replicates (*n* = 3).

Finally, we determined the optimal supply concentration of glycerol as a carbon source in the culture medium. The optimal glycerol concentration was 4%, at which approximately 18.6 mg/l genistein was produced from 300 μM *p*-coumaric acid (Fig. 7). Subsequently, genistein production from *p*-coumaric acid under the optimized conditions was monitored for 72 h. Naringenin production was detected after 4 h, and the production of genistein was first detected after 8 h of biotransformation. Most of the *p*-coumaric acid was converted to naringenin after 24 h. At this point, the production of naringenin reached a maximum. Subsequently, the concentration of naringenin was decreased, while the production of genistein continued to increase. The final yield of genistein was approximately 18.9 mg/l after 72 h of incubation, after which the concentration of genistein decreased slightly (Fig. 8).

Genistein has attracted much attention from researchers because of its various health benefits. In recent years, researchers have attempted to biosynthesize genistein by introducing plant-derived genistein biosynthesis genes into microorganisms [24, 29]. However, several genes must be introduced and functionally expressed in the microorganisms for the complete synthesis of genistein from simple carbon sources such as glucose and glycerol. Although the expression of genes can be controlled by introducing vectors with different copy numbers, it is generally difficult to precisely control their expression [28, 30]. In addition, biotransformation in monoculture requires the supply of various co-factors at each step, which can be difficult to supply at the appropriate concentrations because of competitive use, leading to an increased metabolic burden. Recently, co-culture systems have been designed to produce biologically active compounds using multiple microorganisms instead of a monoculture; this has several advantages, such as avoiding unwanted byproduct formation and minimizing the metabolic burden during biosynthesis [20, 21]. In this study, we attempted to produce genistein from naringenin or *p*-coumaric acid, and optimized the culture system. Under optimized conditions, 35 mg/l and 18.6 mg/l genistein was produced from naringenin and *p*-coumaric acid, respectively, after 48 h of culture. To our knowledge, this is the first study to attempt the biosynthesis of genistein from *p*-coumaric acid. However, to increase the efficiency of genistein production in future, NADPH availability and the stable expression in *E. coli* must be increased through gene codon optimization.

## References

[1] Kaufman PB, Duke JA, Brielmann H, Boik J, Hoyt JE (1997). A comparative survey of leguminous plants as sources of the isoflavones, genistein and daidzein: implications for human nutrition and health. J. Altern. Complement Med..

[2] Morris PF, Ward EWB (1992). Chemoattraction of zoospores of the soybean pathogen, Phytophthora sojae, by isoflavones. Physiol. Mol. Plant Pathol..

[3] Swinny EE, Ryan KG (2005). Red clover *Trifolium pratense* L. phytoestrogens: UV-B radiation increases isoflavone yield, and postharvest drying methods change the glucoside conjugate profiles. J. Agric. Food Chem..

[4] Rivera-Vargas LI, Schmitthenner AF, Graham TL (1993). Soybean flavonoid effects on and metabolism by Phytophthora sojae. Phytochemistry.

[5] Fang K, Dong H, Wang D, Gong J, Huang W, Lu F (2016). Soy isoflavones and glucose metabolism in menopausal women: a systematic review and meta-analysis of randomized controlled trials. Mol. Nutr. Food Res..

[6] Pollard M, Luckert PH (1997). Influence of isoflavones in soy protein isolates on development of induced prostate-related cancers in L-W rats. Nutr. Cancer.

[7] Ko KP (2014). Isoflavones: chemistry, analysis, functions and effects on health and cancer. Asian Pac. J. Cancer Prev..

[8] Lee MJ, Kim JH (2007). Estimated dietary isoflavone intake among Korean adults. Nutr. Res. Pract..

[9] Mora-Pale M, Sanchez-Rodriguez SP, Linhatrdt RJ, Dordick JS, Koffas MAG (2013). Metabolic engineering and in vitro biosynthesis of phytochemicals and non-natural analogues. Plant Sci..

[10] Wang Y, Chen S, Yu O (2011). Metabolic engineering of flavonoids in plants and microorganisms. Appl. Microbiol. Biotechnol..

[11] Han SH, Kim BG, Yoon JA, Chong Y, Ahn JH (2014). Synthesis of flavonoid *O*-pentosides by *Escherichia coli* through engineering of nucleotide sugar pathways and glycosyltransferase. Appl. Environ. Microbiol..

[12] Kim BG (2019). Biosynthesis of bioactive isokaempferide from naringenin in *Escherichia coli*. J. Appl. Biol Chem..

[13] Kim BG, Shin KH, Lee Y, Hur HG, Lim Y, Ahn JH (2005). Multiple regiospecific methylations of a flavonoid by plant Omethyltransferases expressed in E. coli. Biotechnol. Lett..

[14] Ahn BC, Kim BG, Jeon YM, Lee EJ, Lim Y, Ahn JH (2009). Formation of flavone di-*O*-glucosides using a glycosyltransferase from *Bacillus cereus*. J. Microbiol. Biotechnol..

[15] Lee YJ, Kim BG, Park Y, Lim YH, Hur HG, Ahn JH (2006). Biotransformation of flavonoids with *O*-methyltransferase from *Bacillus cereus*. J. Microbiol. Biotechnol..

[16] Yang SM, Han SH, Kim BG, Ahn JH (2014). Production of kaempferol 3-O-rhamnoside from glucose using engineered *Escherichia coli*. J. Ind. Microbiol. Biotechnol..

[17] Lim CG, Fowler ZL, Hueller T, Schaffer S, Koffas MA (2011). High-yield resveratrol production in engineered *Escherichia coli*. Appl. Environ. Microbiol..

[18] Fang Z, Jones JA, Zhou J, Koffas MAG (2018). Engineering *Escherichia coli* co-cultures for production of curcuminoids from glucose. Biotechnol. J..

[19] Chemler JA, Fowler ZL, McHugh KP, Koffas MA (2010). Improving NADPH availability for natural product biosynthesis in *Escherichia coli* by metabolic engineering. Metab. Eng..

[20] Jones JA, Vernacchio VR, Sinkoe AL, Collins SM, Ibrahim MHA, Lachance DM (2016). Experimental and computational optimization of an *Escherichia coli* co-culture for the efficient production of flavonoids. Metab. Eng..

[21] Wang R, Zhao S, Wang Z, Koffas MA (2019). Recent advances in modular co-culture engineering for synthesis of natural products. Curr. Opin. Biotechnol..

[22] Winkel-Shirley B (2001). Flavonoid biosynthesis. A colorful model for genetics, biochemistry, cell biology and biotechnology. Plant Physiol..

[23] Kim BG, Kim SY, Song HS, Lee C, Hur HG, Kim SI (2003). Cloning and expression of the isoflavone synthase gene (IFS-Tp) from *Trifolium pratense*. Mol Cells..

[24] Kim DH, Kim BG, Jung NR, Ahn JH (2009). Production of genistein from naringenin using *Escherichia coli* containing isoflavone synthase-cytochrome P450 reductase fusion protein. J. Microbiol. Biotechnol..

[25] Lee YJ, Jeon Y, Lee JS, Kim BG, Lee CH, Ahn JH (2007). Enzymatic synthesis of phenolic CoAs using 4-coumarate:coenzyme A ligase (4CL) from rice. Bull Korean Chem. Soc..

[26] Kim BG, Lee ER, Ahn JH (2012). Analysis of flavonoid contents and expression of flavonoid biosynthetic genes in Populus euramericana Guinier in response to abiotic stress. J. Korean Soc. Appl. Biol. Chem..

[27] Jones JA, Vernacchio VR, Lachance DM, Lebovich M, Fu L, Shirke AN (2015). ePathOptimize: a combinatorial approach for transcriptional balancing of metabolic pathways. Sci. Rep..

[28] Jones JA, Collins SM, Vernacchio VR, Lachance DM, Koffas MAG (2016). Optimization of naringenin and *p*-coumaric acid hydroxylation using the native *E. coli* hydroxylase complex, HpaBC. Biotechnol. Prog..

[29] Leonard E, Koffas MAG (2007). Engineering of artificial plant cytochrome P450 enzymes for synthesis of isoflavones by *Escherichia coli*. Appl. Environ. Microbiol..

[30] Kim MJ, Kim BG, Ahn JH (2013). Biosynthesis of bioactive O-methylated flavonoids in *Escherichia coli*. Appl. Microbiol. Biotechnol..

